# Circulating miR-185-5p as a Potential Biomarker for Arrhythmogenic Right Ventricular Cardiomyopathy

**DOI:** 10.3390/cells10102578

**Published:** 2021-09-28

**Authors:** Claudia Sacchetto, Zenab Mohseni, Robin M. W. Colpaert, Libero Vitiello, Marzia De Bortoli, Indira G. C. Vonhögen, Ke Xiao, Giulia Poloni, Alessandra Lorenzon, Chiara Romualdi, Riccardo Bariani, Elisa Mazzotti, Luciano Daliento, Barbara Bauce, Domenico Corrado, Thomas Thum, Alessandra Rampazzo, Leon J. de Windt, Martina Calore

**Affiliations:** 1 Department of Molecular Genetics, Faculty of Science and Engineering, Faculty of Health, Medicine and Life Sciences, Maastricht University, 6229 ER Maastricht, The Netherlands; c.sacchetto@maastrichtuniversity.nl (C.S.); zenab1405@gmail.com (Z.M.); R.Colpaert@maastrichtuniversity.nl (R.M.W.C.); igcvonhogen@me.com (I.G.C.V.); L.deWindt@maastrichtuniversity.nl (L.J.d.W.); 2 Department of Biology, Padova University, 35121 Padova, Italy; libero.vitiello@bio.unipd.it (L.V.); Marzia.DeBortoli@eurac.edu (M.D.B.); giuliapoloni7@gmail.com (G.P.); alessandra.lorenzon7@gmail.com (A.L.); chiara.romualdi@unipd.it (C.R.); alessandra.rampazzo@unipd.it (A.R.); 3 Eurac Research, Institute for Biomedicine, University of Lübeck, 39100 Bolzano, Italy; 4 Institute of Molecular and Translational Therapeutic Strategies (IMTTS), Hannover Medical School, D-30625 Hannover, Germany; Xiao.Ke@mh-hannover.de (K.X.); thum.thomas@mh-hannover.de (T.T.); 5 Fraunhofer Institute for Toxicology and Experimental Medicine (ITEM), 30625 Hannover, Germany; 6 Department of Cardiac, Thoracic, and Vascular Sciences, Padova University, 35121 Padova, Italy; riccardo.bariani@unipd.it (R.B.); elymazzotti@yahoo.com (E.M.); luciano.daliento@unipd.it (L.D.); barbara.bauce@unipd.it (B.B.); domenico.corrado@unipd.it (D.C.); 7 CRIBI Biotechnology Centre, University of Padova, 35121 Padova, Italy

**Keywords:** arrhythmogenic right ventricular cardiomyopathy, MicroRNAs, circulating microRNAs, heart failure, biomarkers, genetics

## Abstract

Arrhythmogenic right ventricular cardiomyopathy (ARVC) is a genetic cardiac disease characterized by progressive myocardial fibro-fatty replacement, arrhythmias and risk of sudden death. Its diagnosis is challenging and often it is achieved after disease onset or postmortem. In this study, we sought to identify circulating microRNAs (miRNAs) differentially expressed in ARVC patients compared to healthy controls. In the pilot study, we screened the expression of 754 miRNAs from 21 ARVC patients and 20 healthy controls. After filtering the miRNAs considering a log fold-change cut-off of ±1, *p*-value < 0.05, we selected five candidate miRNAs for a subsequent validation study in which we used TaqMan-based real-time PCR to analyse samples from 37 ARVC patients and 30 healthy controls. We found miR-185-5p significantly upregulated in ARVC patients. Receiver operating characteristic analysis indicated an area under the curve of 0.854, corroborating the link of this miRNA and ARVC pathophysiology.

## 1. Introduction

Among the causes of heart failure, arrhythmogenic right ventricular cardiomyopathy (ARVC) is a genetic cardiac disease characterized by progressive myocardial loss and fibro-fatty replacement [1,2]. It is the second most common cause of sudden death in the young, after hypertrophic cardiomyopathy, and may account for as many as 22.4% of heart failure events among athletes [3,4]. To date, 18 ARVC genes have been discovered, with 40% of mutations identified in three major genes, encoding for plakophilin-2 (PKP2), desmoplakin (DSP), and desmoglein-2 (DSG2), which affect desmosomal and area composita proteins, the mechanical cell junctions in the cardiac intercalated disc [5]. The diagnosis of ARVC relies on the combination of major and minor criteria established by a specific task force, including electrical, structural, electrophysiological and genetic factors, and can be very challenging [6]. Indeed, ARVC is often established postmortem, highlighting the urgent need to identify novel disease-specific biomarkers for the early detection of this devastating disease.

MicroRNAs (miRNAs) are a class of short non-coding RNAs that regulate gene expression at the post-transcriptional level and are involved in a wide range of physiological processes [7]. Extracellular miRNAs can circulate and are stable in the biofluids, including plasma, of both animals and humans [8,9], either within membranous vesicles or associated with RNA-binding proteins [10]. For this reason, and due to their implication in several cardiac diseases, miRNAs have recently attracted particular interest as potential non-invasive biomarkers [11]. So far, few studies have evaluated circulating miRNAs differentially expressed in the heart and in plasma of ARVC patients, showing conflicting results [12,13,14,15].

In this study, we aimed to profile circulating miRNAs in a cohort of 37 ARVC patients. We found miR-185-5p to be significantly altered in the plasma of ARVC patients, confirming its correlation with the disease. 

## 2. Materials and Methods

### 2.1. Patients’ Cohort and Clinical Evaluation

The study population consisted of 37 unrelated patients (22 males and 15 females, mean age at diagnosis 44 ± 13) diagnosed with ARVC according to 2010 Task Force criteria [6] (Supplementary Figure S1 and Table S1) and 30 unrelated, age- and sex-matched healthy controls (HCs). All clinical investigations were conducted according to the principles expressed in the Declaration of Helsinki; written informed consent was obtained from all participants. The study was approved by the Institutional Committee on Human Research at the authors’ institution (project identification code: 38083). Clinical evaluation consisted of a detailed personal/family history, physical examination, 12-lead electrocardiogram (ECG), signal-averaged (SAECG), 24-h Holter ECG, and 2-dimensional echocardiography (2D-echo). Contrast-enhanced cardiac magnetic resonance (CMR) was performed according to previously reported methods [16].

### 2.2. Plasma Isolation and RNA Extraction

For plasma isolation, total blood was collected in Sarstedt S-Monovette “EDTA Kalium-Gel”, 7.5 mL (Sarstedt #01.1621.001) according to the manufacturer’s instructions. The first 3 mL of blood were discarded to prevent contamination with skin-derived material while priming the interior volume of the blood collection set. The tubes were then thoroughly mixed by inversion 10 times to ensure homogenous mixing with anticoagulant. Plasma isolation was performed by centrifuging the tubes in a swing-out centrifuge at 2.500× *g* for 15 min at room temperature. Supernatant plasma was then collected, aliquoted, and stored at −80 °C until processing.

Total RNA was extracted from 100 uL of plasma using the miRNeasy Serum/Plasma Kit (Qiagen, Cat No. 217184), according to the manufacturer’s protocol.

### 2.3. Pilot Study

**Figure 1:**
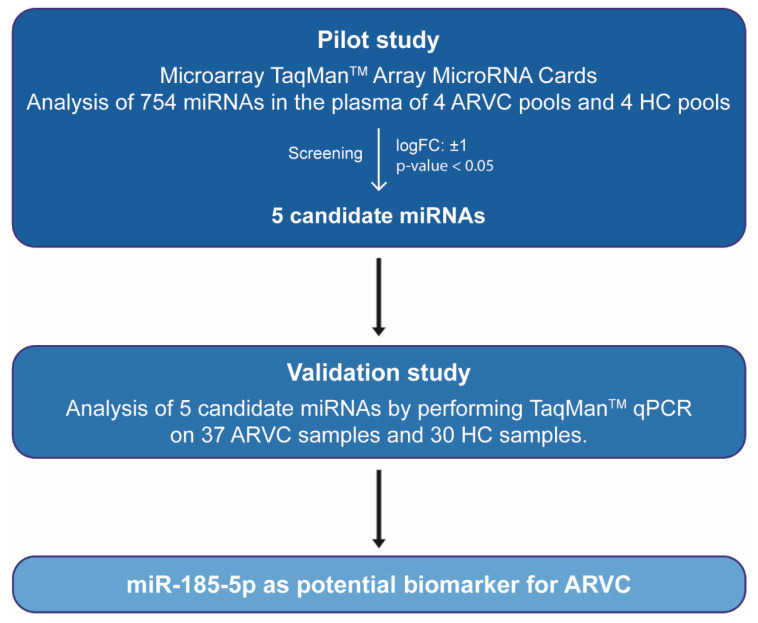
Workflow of the study demonstrating the screening process used to determine the miRNAs altered in ARVC patients. ARVC, Arrhythmogenic Right Ventricular Cardiomyopathy; HC, healthy controls; miRNAs, microRNAs; qPCR, quantitative polymerase chain reaction.

Taqman Array Human MicroRNA A + B Cards Set version 3.0 (Life Technologies, Cat No. 4444913) was used to analyse the expression level of 754 circulating miRNAs in 21 ARVC patients and 20 healthy controls (HC) (Figure 1) following the manufacturer’s recommendation. Prior to polymerase chain reactions (PCR), two steps consisting of reverse-transcription (RT) and preamplification were conducted. The Megaplex (Applied Biosystems) PreAmp Primers, consisting of two pools of gene-specific forward and reverse primers (Pool A and Pool B) were used to enable the unbiased preamplification of the miRNA cDNA target by PCR.

The ARVC samples were pooled according to the patients’ genotype or phenotype, as follows: samples from five ARVC patients carrying a frameshift mutation in the *PKP2* gene were grouped in pool 1; samples from five patients with a severe phenotype that had required the implantation of the cardioverter defibrillator were collected in pool 2; samples from six patients carrying variants in the desmosomal genes *DSP* and *DSG2* were grouped in pool 3, and samples from five more ARVC patients were grouped in pool 4 without making a distinction on the genotype or on a particular phenotypic feature. Samples from HCs were grouped in 4 pools of 5 individuals each in a random fashion. Data were analysed with QuantStudio v.1.3 (Life Technologies) and ExpressionSuite v1.0.3 (Life Technologies), followed by both global normalization and endogenous control normalization. For the latter, miR-484 and miR-93-5p were chosen among miRNAs known to be stably expressed in plasma that were present in the Taqman Megaplex panel (hsa-miR-24, hsa-miR-484, hsa-mir-93-5p, hsa-miR-191-5p, hsa-miR-126-3p, hsa-miR-16) by using BestKeeper software https://www.gene-quantification.de/bestkeeper.html (accessed on 1 December 2017) based on very limited intra-group variability expression [17]. Data obtained from all normalization methods were reproducible, therefore we chose hsa-miR-484 (Cat. No. A25576) as normalizer in the following steps.

### 2.4. Validation Study

In the validation study, 16 additional patients and 10 control samples were added to the cohort, which now included a total of 37 ARVC and 30 HC individuals (Figure 1). TaqMan Advanced miRNA Assays (Life Technologies, Cat No. A28007) was used for the evaluation of expression levels of the five candidate miRNAs in the individual samples, following manufacturer’s instructions. All samples were evaluated in triplicates, considering a threshold cycle (Ct) < 35, and a ± 0.5 maximal difference between the detected Ct values. Relative quantification was obtained using the 2^−ΔΔCt^ method, using miR-484 as endogenous control for normalization. 

### 2.5. Target Prediction and Pathway Analysis

For target prediction of differentially expressed miRNAs, the DNA Intelligent Analysis (DIANA)-micro T-CDS v5.0 prediction algorithm, the DIANA-miRPath v.3 algorithm and the microRNA—target interaction database MiRTarBase were used [18,19,20]. Among the predicted and validated target genes, we focused on those bearing a clear relevance for the disease, i.e., those encoding for components of cardiac mechanical and electrical junctions, as well as those involved in the Wnt and Hippo pathways, which are often found altered in ARVC [21,22,23].

### 2.6. Statistical Analysis

Patient baseline characteristics are described as mean (standard deviation) and absolute number (percentage). Data for miRNA expression levels are reported as mean values ± standard error (SE). Categorical values are reported as absolute values and percentages. The Mann-Whitney U test and Kruskal Wallis test were used for the comparison of data without the assumption of a normal distribution. *p*-value < 0.05 was considered statistically significant. Moreover, the area under the curve (AUC) of receiver-operating characteristic (ROC) plots was used to assess miRNA diagnostic accuracy as a biomarker for ARVC. The best cut-off values for the ROC-curves were calculated via the Youden index (J). Statistical analyses were performed using SPSS Statistics version 21.0 (IBM Corp., Armonk, NY, USA), GraphPad PRISM Version 7, and R version 3.4.4. 

## 3. Results

### 3.1. Identification of Circulating miRNAs Differentially Expressed in ARVC Patients

**Figure 2:**
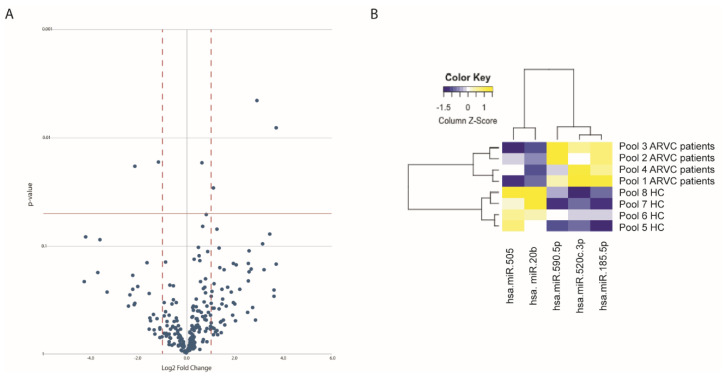
Circulating miRNAs differentially expressed in ARVC patients. (**A**) Volcano plot showing the differential expression of the circulating miRNAs in ARVC patients. Dots above the *p*-value = 0.05 threshold line (red solid line) indicate statistically significant dysregulated miRNAs. Red dashed lines indicate the LogFC threshold (±1). (**B**) Heatmap reporting a visual representation of the five candidate miRNAs differentially expressed in ARVC pools compared with HC groups.

In the pilot study, the expression level of 754 circulating miRNAs was analysed in 21 ARVC patients and 20 healthy controls; both cohorts were divided into four pools, each comprising five individuals, except for pool 3, which had six patients. Data were analysed with QuantStudio v.1.3 and ExpressionSuite v1.0.3 followed by both global normalization and endogenous control normalization (using either miR-484 or miR-93-5p as endogenous control), obtaining reproducible results. Out of 754 miRNAs, 240 resulted expressed in the plasma (Supplementary Table S2). While no significant differences were detected in the circulating miRNA expression among the different patient pools, five circulating miRNAs showed a significant differential expression between ARVC patients and healthy controls and were considered in the validation study (Figure 2A,B, Table 1 and Supplementary Table S2). Two of them (miR-20b and miR-505) were significantly down-regulated in patients compared to controls, while three miRNAs (miR-520c-3p, miR-590-5p, and miR-185-5p) were upregulated in the ARVC cohort (Table 1).

### 3.2. Validation of Circulating miRNAs Differentially Expressed in ARVC Patients

**Figure 3:**
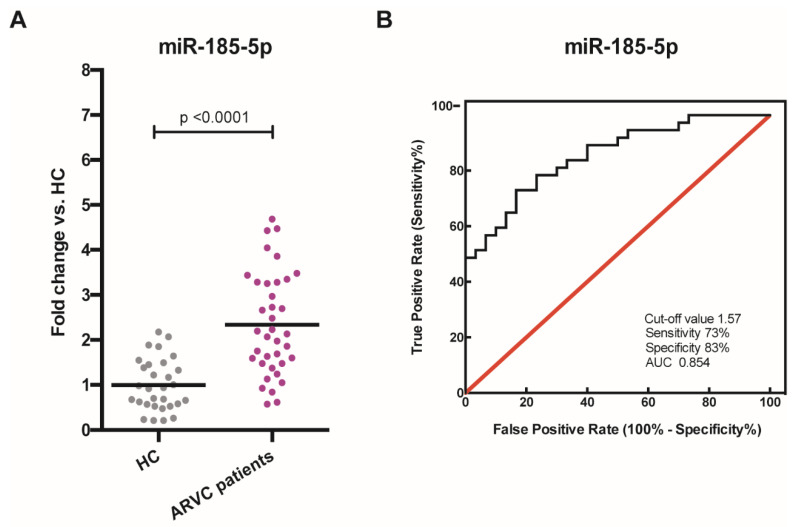
Validation of miRNA dysregulation in ARVC patients and predictive role of miR-185-5p. (**A**) Scatterplot showing miR-185-5p upregulation in ARVC patients (*n* = 37) compared with HC (*n* = 30). HC = healthy controls, ARVC = arrhythmogenic cardiomyopathy. (**B**) Receiver operating characteristic (ROC) analysis of miR-185-5p: cut-off value 1.57 (fold change; AUC: 0.854). AUC = area under the curve.

The five candidate miRNAs were considered for individual RT-qPCRs in the larger cohorts of 37 ARVC samples and 30 HC (which partially overlaps with the respective discovery cohorts). This validation study confirmed the increased expression for miR-185-5p (2.34 ± 0.19, *p*-value < 0.0001, Figure 3A, Supplementary Table S3) in ARVC patients compared to HC. In contrast, exceptions were made for miR-505, miR-520c-3p, and miR-590-5p that could not be amplified by the respective primers; miR-20b resulted significantly downregulated but to a lower extent compared to the pilot study (0.55 ± 0.19, *p*-value < 0.001, Supplementary Figure S2), and therefore it was not considered further. 

Next, in order to assess the potential diagnostic utility of miR-185-5p as a candidate biomarker for ARVC in our cohort, we plotted the receiver-operating characteristic (ROC) curve and calculated the relative area under the curve (AUC) (Figure 3B), which was to 0.854. The best cut-off value, estimated through the Youden Index method, was 1.57 (fold change).

### 3.3. In Silico Analyses

In silico analyses on DIANA mirPath v.3, DIANA-micro T-CDS v.5.0 and MiRTarBase suggest the involvement of miR-185-5p in the regulation of cell adhesion, notably altered in ARVC, and in Wnt and Hippo pathways, which have already been shown to be implicated in ARVC pathogenesis (Table 2) [21,22,23].

## 4. Discussion

ARVC diagnosis is particularly difficult: it relies on the combination of major and minor task force criteria, it can imply invasive procedures (e.g., endomyocardial biopsy) and it is often established postmortem [1,2,3]. Novel tools helping in the diagnosis of this disease are thus urgently required. HereIn this study we found miR-185-5p significantly altered in the plasma of 37 ARVC patients. Our findings partially overlap with those of Yamada and colleagues, who recently analysed the expression of 84 cardiac-related miRNAs in the plasma of 28 ARVC patients and reported a significant upregulation of four miRNAs, including miR-185-5p [14]. Altogether, Yamada’s study and ours found this miRNA overexpressed in a total of 65 patients, further confirming the potential role of miR-185-5p as a circulating biomarker for ARVC. Of note, the same study excluded alterations on miR-185-5p in patients affected with idiopathic ventricular tachycardia [14]. Interestingly, miR-185-5p was not significantly altered in the plasma of patients affected with cardiac amyloidosis [24], dilated cardiomyopathy [25], or chronic heart failure [26], analysed using our same technical approach, thus corroborating the specificity of this miRNA’s dysregulation in ARVC. Moreover, it is interesting to note that alterations in miR-185 were excluded in large-scale studies on patients affected with other cardiac diseases, such as inflammatory heart disease [27] and myocardial fibrosis [28]. Interestingly, in silico analyses suggest that deregulation in miR-185-5p might result in perturbed cellular functions and pathways critically altered in ARVC. In particular, impaired mechanical and electrical junctions are frequently detected in ARVC and are thought to contribute to cardiomyocyte death and electrical alterations that are typical of the disease [29]. Moreover, abnormal inhibition of the Wnt signalling and activation of the Hippo pathway have been reported in different ARVC models [21,22,23]. If, on the one hand, these data further support the involvement of circulating miR-185-5p in ARVC, on the other hand functional studies on relevant disease models will be required to dissect the detailed interactions among the miRNA and the selected genes.

Besides miR-185-5p, Yamada et al. found miR-144-3p, miR-145-5p and miR-494 upregulated in ARVC [14]. Sommariva et al. analysed the expression of 377 miRNAs in the plasma of 36 ARVC patients, reporting that miR-320a was significantly downregulated [13]. More recently, Bueno Marinas et al. reported miR-149-5p, miR-494-3p, and miR-144-5p to be reduced in the plasma of ARVC patients [15]. The fact that we did not find alterations in the expression of these miRNAs in our cohort might be due to the different normalization methods used in the studies. Specifically, in our pilot study we applied an endogenous control (using miR-484 or miR-93 as normalizers) as well as global normalization; in the validation study, we chose miR-484 as normalizer because it showed the lowest intra-group variability. On the other hand, the studies by Yamada et al. and Bueno Marinas et al. used *C.el*-miR-39 as normalizer, while Sommariva et al. adopted miR-210 [13,14,15]. Also of notice, our validation approach is different compared to the ones chosen in the aforementioned studies; in fact, we used TaqMan probes in order to avoid any possible bias related to the non-specific amplification of miRNAs with similar sequences, which may occur when using standard primer sets. On the contrary, in Yamada et al., and in Bueno Marinas et al., the validation study was performed using standard primer pairs [14,15].

On the other hand, the fact that we and Yamada et al. found miR-185-5p upregulated in ARVC patients using different approaches strongly supports this result beyond the technical bias. This is the first time that a circulating miRNA shows the same trend in different studies performed in different ARVC cohorts, confirming the potential of circulating miR-185-5p as a useful biomarker in establishing ARVC diagnosis. Still, given the non-complete correspondence with other similar studies, it will be important to further evaluate the expression of this miRNAin the cohorts of different patients, ideally comprising patients showing different genotypes and/or specific features of the disease. In addition, expanding validation studies into geographically independent ARVC cohorts would help avoiding any potential bias related to founder effects in the genetic background. Lastly, it is important to highlight that the highly desirable goal of validating specific miRNAs as ARVC biomarkers would greatly benefit from the definition of specific standard protocols by the ARVC research community.

### Study Limitations

While our findings point towards a possible role of circulating miR-185-5p as a biomarker for ARVC, the present study presents some design limitations that should be considered before drawing a firm conclusion. Firstly, although previous studies have excluded the involvement of circulating miR-185-5p in other cardiac diseases [14,24,25,26,27,28], in order to conclusively determine that its upregulation is specific to ARVC, our experimental approach will have to also be applied to patients affected with other cardiac diseases, such as coronary or congenital heart disorders, as well as other forms of arrhythmia. Secondly, the discovery cohort was a subset of the validation cohort and hence the latter will need to be extended to eliminate the overlap in order to avoid any potential bias in the results.

## Figures and Tables

**Table 1:** List of circulating miRNAs differentially expressed in ARVC patients.

miRNA	LogFC	*p*-Value
hsa-miR-505	−1.139	0.017134
hsa-miR-20b	−2.112	0.018686
hsa-miR-590-5p	1.117	0.029870
hsa-miR-520c-3p	3.716	0.008194
hsa-miR-185-5p	2.931	0.004594

**Table 2:** List of predicted and experimentally validated targets of miR-185-5p.

**miR-185-5p**	**Target Genes**			
	**Adherens** **Junctions**	**Gap** **Junctions**	**Wnt** **Pathway**	**Hippo** **Pathway**
	LMO7, IQGAP1, EGFR, RHOA *, TJP1, MLLT4, PTPN6, PTPRJ, CDC42	ADCY2, TJP1, PRKG2, PRKCB, GJA1, ADCY4	CCND2, CDC42 *, CCNE1 *, CDK6 *, AKT1 *, HMGA1 *, HMGA2 *, SIX1 *, DNMT1 *, EPAS1 *, SCARB1 *, TP53, PPP1CC, CAM4K *, CAMKK2, WNT5B, EZH2 *, NFATC3 *, CDK14, LRP3, CNTNAP2, SMAD7 *, TL3, GSK3β, CCND1	GSK3β, CCND1, CCND2, YWHAE, YWHAG, YWHAB, WWTR1, YWHAQ, CSNK1D, DLG4, TEAD1, CSNK1E, MOB1A, PPP2R1B, TJP1, AMOTL2

* Genes that were experimentally validated as targets of miR-185-5p. In silico analyses were performed on DIANA mirPath v.3, DIANA-micro T-CDS v.5.0 and MiRTarBase.

## Data Availability

The data presented in this study are available in supplementary material.

## Supplementary Materials

The following are available online at https://www.mdpi.com/article/10.3390/cells10102578/s1, Figure S1: Electrocardiographic and cardiac magnetic resonance features of a representative ARVC patient. Figure S2: Validation of miR-20b dysregulation in ARVC patients. Table S1: Clinical data of ARVC patients. Table S2: MiRNA screening data. Table S3: Expression level of miR-185-5p in the validation cohort.
