# Supplementary material for: Circulating miR-185-5p as a Potential Biomarker for Arrhythmogenic Right Ventricular Cardiomyopathy
Source: Cells. 2021 Sep 28;10(10):2578. doi: 10.3390/cells10102578 (PMC8533962; doi:10.3390/cells10102578)
Supplement: Supplementary file 1 [file cells-10-02578-s001.zip › cells-1364485-supplementary.pdf]

Supplementary Materials

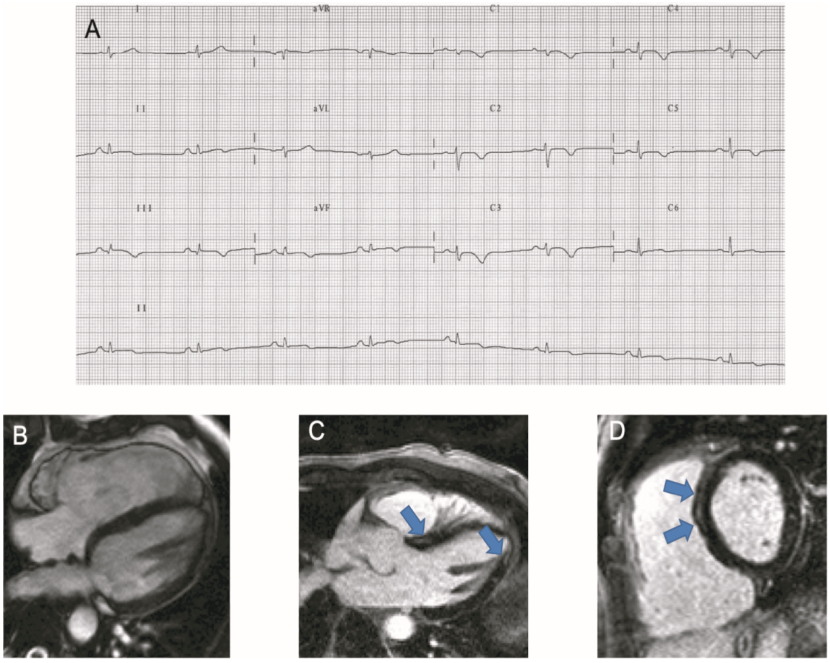

Figure S1. **Electrocardiographic and cardiac magnetic resonance features of a representative ARVC patient. (A)** Twelve-lead ECG showing sinus rhythm, T wave inversion in V1-V5, aVF, DIII, low QRS voltages in peripheral leads suggesting a biventricular involvement. **(B)** CMR SSFP sequence showing a severe RV dilatation and disfunction. Several bulges are present in the subtricuspidalic area. **(C)** and **(D)** CMR post contrast sequences showing late gadolinium enhancement (LGE, blue arrows) in anterior septum and apex. LGE presence is confirmed in a short axis view (**D**).

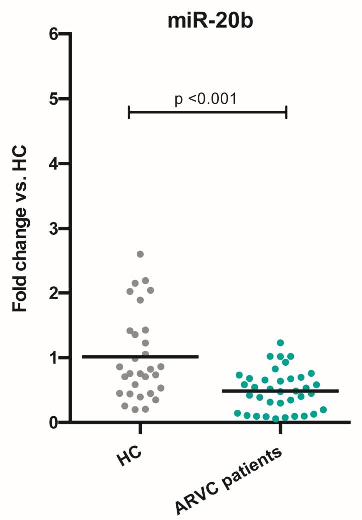

Figure S2. Validation of miR-20b dysregulation in ARVC patients. Scatterplot showing miR-20b downregulation in ARVC patients (n=37) compared with HC (n=30). HC= healthy controls, ARVC= arrhythmogenic cardiomyopathy.

Table S1. Clinical data of ARVC patients.

Patient	Gen-der	Age (yrs) at Diagnosis/ Last Investigation	Family		Echocardiography RV Global or Regional Dysfunction and Structural Alterations		Twelve-lead ECG Depolarization Abnormalities			Twelve-lead ECG Depolarization/ Conduction Abnormalities		Arrhythmias			LV Involved	ARVC Diagnostic Criteria
			(M)	(m)	Regional RV akinesia, dyskinesia, or aneurysm and 1 of the following (end diastole): (M)<br>PLAX RVOT ≥32 mm; PSAX RVOT ≥36 mm; FAC ≤33%	Regional RV akinesia, dyskinesia, or aneurysm and 1 of the following (end diastole): (m)<br>PLAX RVOT ≥29 to <32 mm; PSAX RVOT ≥32 to <36 mm; FAC >33% to ≤40%	Inverted T waves in V1-V3 (>14 yrs) (M)	Inverted T waves in V1-V2 (>14 yrs) or in V4-V6 (m)	Inverted T waves in V1-V4 (>14 yrs) in presence of RBBB (m)	Epsilon wave (M)	Late potentials (SAECG) (m)	NSVT or SVT of LBBB morphology with superior axis (M)	NSVT or SVT of RV outflow configuration or of unknown axis (m)	>500 ventricular extrasistoles per 24 hours (holter) (m)		
3	M	55	0	0	0	1	1	0	0	0	0	1	0	0	1	2M, 1m
5	F	32	0	0	1	0	0	0	1	0	0	1	0	0	0	2M,1m
7	F	38	1	0	1	0	1	0	0	0	0	0	0	1	1	3M, 1m
9	M	49	0	0	1	0	1	0	0	0	1	0	0	1	0	2M, 2m
11	M	40	0	1	1	0	1	0	0	0	0	0	0	1	1	2M, 2m
15	F	43	1	0	1	0	1	0	0	0	1	0	0	1	0	3M, 2m
17	M	48	1	0	1	0	0	1	0	0	0	1	0	1	0	2M, 3m
22	M	14	0	0	1	0	1	0	0	0	1	0	0	1	0	2M, 2m

24	F	59	1	0	0	1	0	0	0	0	1	1	0	0	1	2M,2m
27	F	48	0	1	0	1	1	0	0	0	1	0	0	1	0	1M, 4m
32	M	59	1	0	1	0	1	0	0	0	1	0	1	0	1	3M, 2m
35	M	38	1	0	1	0	1	0	0	0	0	0	0	1	0	3M, 1m
45	M	47	0	0	0	1	1	0	0	0	0	1	0	1	0	2M, 2m
46	M	33	0	1	1	0	0	1	0	0	0	0	0	1	1	1M, 3m
47	F	32	0	0	1	0	1	0	0	0	0	1	0	0	1	3M
55	M	58	0	0	1	0	1	0	0	0	1	0	1	0	1	2M,2m
56	M	31	0	1	1	0	1	0	0	0	0	0	0	1	0	2M, 2m
57	M	36	0	0	0	1	0	1	0	0	1	0	0	1	0	4m
60	M	39	1	0	0	1	0	0	0	0	1	0	0	1	0	1M, 3m
62	M	46	1	0	1	0	1	0	0	0	0	0	0	1	0	3M, 1m
63	M	64	0	0	0	1	0	1	0	0	1	0	1	0	1	4m
92	F	35	0	1	0	1	0	1	0	0	0	1	0	1	0	4m
93	F	54	0	0	0	1	1	0	0	0	1	0	0	1	0	1M,3m
94	M	58	0	0	1	0	1	0	0	0	0	0	1	0	0	2M, 1m
95	F	63	0	0	0	1	0	1	0	0	1	0	0	1	1	4m
97	F	28	1	0	0	1	1	0	0	0	0	0	0	0	1	1M, 2m

99	F	45	0	1	0	1	1	0	0	0	1	0	0	1	0	1M, 4m
100	F	16	1	0	1	0	1	0	0	0	1	0	0	1	1	3M, 2m
102	M	34	0	0	0	1	0	1	0	0	1	0	0	1	0	4m
103	M	71	1	0	0	0	0	1	0	0	1	0	0	0	1	1M,2m
105	F	49	1	0	1	0	1	0	0	0	1	0	0	1	1	3M, 2m
107	M	52	0	0	0	1	0	1	0	0	1	0	0	1	0	4m
109	F	45	0	0	1	0	1	0	0	0	1	1	0	0	1	3M,1m
114	M	33	1	0	0	1	0	1	0	0	1	1	0	0	1	2M, 3m
115	M	41	1	0	1	0	1	0	0	0	1	0	0	1	1	3M, 2m
120	M	59	0	1	1	0	1	0	0	0	1	0	0	1	0	2M 3m
121	F	34	0	1	1	0	1	0	0	0	0	0	0	1	0	2M, 2m

ARVC: arrhythmogenic right ventricular cardiomyopathy; FAC: fractional area change; LBBB: left bundle branch block; LV: left ventricle; M/m: major/minor diagnostic criterion; NSVT/SVT: not-sustained/sustained ventricular tachycardia; PLAX: parasternal long-axis view; PSAX: parasternal short-axis view; RV: right ventricle; RVOT: RV outflow tract; 1: present; 0: absent.

Table S2. miRNA screening data.

miRNA	Ct values (mean)								Δ Ct values *								Log FC	p-value
	ARVC (N = 21)				HC (N = 20)				ARVC (N = 21)				HC (N = 20)					
	Pool 1	Pool 2	Pool 3	Pool 4	Pool 5	Pool 6	Pool 7	Pool 8	Pool 1	Pool 2	Pool 3	Pool 4	Pool 5	Pool 6	Pool 7	Pool 8		
hsa-miR-99b#-002196	27.984	29.869	6.659	31.827	33.153	30.932	30.939	28.651	8.873	7.070	6.659	10.550	10.996	9.687	9.784	5.452	-0.692	0.6637
hsa-miR-99b-000436	25.057	31.888	6.509	28.141	28.216	28.306	29.674	31.295	5.946	9.089	6.509	6.864	6.059	7.061	8.519	8.096	-0.332	0.7203
hsa-miR-99a-000435	27.809	29.839	9.559	30.595	29.946	28.941	29.948	28.885	8.698	7.040	9.559	9.318	7.789	7.696	8.793	5.686	1.163	0.2277
hsa-miR-942-002187	27.847	33.398	11.281	30.141	30.448	29.477	31.492	34.640	8.736	10.599	11.281	8.864	8.291	8.232	10.337	11.441	0.295	0.7815
hsa-miR-939-002182	30.098	30.780	13.165	31.858	32.136	30.973	30.643	29.877	10.987	7.981	13.165	10.581	9.979	9.728	9.488	6.678	1.710	0.2448
hsa-miR-93#-002139	26.651	30.038	7.435	29.109	28.731	27.908	28.396	30.172	7.540	7.239	7.435	7.832	6.574	6.663	7.241	6.973	0.649	0.0173
hsa-miR-92a-000431	20.221	22.276	-0.143	20.898	22.365	21.185	21.560	23.592	1.110	-0.523	-0.143	-0.379	0.208	-0.060	0.405	0.393	-0.220	0.6050
hsa-miR-892b-002214	30.844	31.117	10.167	35.104	30.765	30.588	30.070	27.739	11.733	8.318	10.167	13.827	8.608	9.343	8.915	4.540	3.160	0.0983
hsa-miR-886-5p-002193	29.088	40	8.423	30.959	34.611	30.657	31.982	32.997	9.977	17.201	8.423	9.682	12.454	9.412	10.827	9.798	0.698	0.7578
hsa-miR-886-3p-002194	29.187	30.812	10.642	29.255	31.027	31.064	31.179	30.124	10.076	8.013	10.642	7.978	8.870	9.819	10.024	6.925	0.268	0.7958
hsa-miR-885-5p-002296	25.735	26.960	4.007	26.015	26.209	26.298	26.860	29.304	6.624	4.161	4.007	4.738	4.052	5.053	5.705	6.105	-0.346	0.6618
hsa-miR-875-5p-002203	31.051	29.329	8.842	31.641	32.706	30.161	29.603	28.815	11.940	6.530	8.842	10.364	10.549	8.916	8.448	5.616	1.037	0.5270
hsa-miR-874-002268	28.376	33.432	8.797	33.614	30.415	32.800	30.564	32.712	9.265	10.633	8.797	12.337	8.258	11.555	9.409	9.513	0.574	0.6045
hsa-miR-769-5p-001998	27.337	39.385	11.578	33.978	32.342	31.750	31.132	33.850	8.226	16.586	11.578	12.701	10.185	10.505	9.977	10.651	1.943	0.3420
hsa-miR-766-001986	23.101	31.268	5.270	27.729	27.344	27.431	27.660	29.052	3.990	8.469	5.270	6.452	5.187	6.186	6.505	5.853	0.113	0.9159
hsa-miR-758-001990	28.272	32.932	8.664	31.427	33.799	31.881	34.240	32.389	9.161	10.133	8.664	10.150	11.642	10.636	13.085	9.190	-1.611	0.1452
hsa-miR-744-002324	22.848	35.752	6.679	28.582	27.224	27.370	28.096	29.986	3.737	12.953	6.679	7.305	5.067	6.125	6.941	6.787	1.438	0.5142

hsa-miR-720-002895	22.488	24.651	2.193	24.837	24.657	23.943	24.529	24.240	3.377	1.852	2.193	3.560	2.500	2.698	3.374	1.041	0.342	0.6176
hsa-miR-708-002341	29.321	27.991	8.158	28.979	30.223	29.233	29.613	29.552	10.210	5.192	8.158	7.702	8.066	7.988	8.458	6.353	0.099	0.9342
hsa-miR-671-3p-002322	26.717	40	8.120	32.257	34.744	31.088	29.571	31.391	7.606	17.201	8.120	10.980	12.587	9.843	8.416	8.192	1.217	0.6407
hsa-miR-664-002897	25.300	40	11.092	32.645	29.982	31.076	33.915	37.391	6.189	17.201	11.092	11.368	7.825	9.831	12.760	14.192	0.310	0.9119
hsa-miR-661-001606	25.588	29.221	5.572	27.800	30.346	26.988	28.129	28.680	6.477	6.422	5.572	6.523	8.189	5.743	6.974	5.481	-0.348	0.6283
hsa-miR-660-001515	25.044	28.392	4.719	26.532	25.909	25.359	25.308	29.494	5.933	5.593	4.719	5.255	3.752	4.114	4.153	6.295	0.797	0.2753
hsa-miR-659-001514	33.243	30.806	9.051	40	35.101	32.310	34.983	29.478	14.132	8.007	9.051	18.723	12.944	11.065	13.828	6.279	1.449	0.6477
hsa-miR-652-002352	24.528	30.758	7.783	28.882	28.117	29.092	28.213	30.958	5.417	7.959	7.783	7.605	5.960	7.847	7.058	7.759	0.035	0.9639
hsa-miR-645-001597	31.976	32.232	9.177	33.197	32.890	33.485	32.163	31.474	12.865	9.433	9.177	11.920	10.733	12.240	11.008	8.275	0.285	0.8253
hsa-miR-642-001592	30.230	32.378	13.098	33.565	32.651	32.912	33.124	40	11.119	9.579	13.098	12.288	10.494	11.667	11.969	16.801	-1.212	0.4825
hsa-miR-639-001583	29.163	30.463	8.673	30.452	30.965	30.587	29.350	28.013	10.052	7.664	8.673	9.175	8.808	9.342	8.195	4.814	1.101	0.3825
hsa-miR-638-001582	29.838	29.086	8.453	40	31.128	30.506	30.219	29.897	10.727	6.287	8.453	18.723	8.971	9.261	9.064	6.698	2.549	0.4214
hsa-miR-636-002088	28.137	31.334	6.828	27.911	30.515	27.909	28.272	30.441	9.026	8.535	6.828	6.634	8.358	6.664	7.117	7.242	0.410	0.5839
hsa-miR-629-001562	31.611	40	10.582	31.833	32.042	33.736	31.432	40	12.500	17.201	10.582	10.556	9.885	12.491	10.277	16.801	0.346	0.8817
hsa-miR-628-5p-002433	26.078	29.356	5.665	27.109	28.036	27.436	23.987	29.212	6.967	6.557	5.665	5.832	5.879	6.191	2.832	6.013	1.026	0.2998
hsa-miR-628-3p-002434	26.063	33.868	7.908	35.220	34.447	34.633	30.205	32.748	6.952	11.069	7.908	13.943	12.290	13.388	9.050	9.549	-1.101	0.5876
hsa-miR-625#-002432	24.117	30.270	6.585	29.129	29.061	28.710	28.818	30.027	5.006	7.471	6.585	7.852	6.904	7.465	7.663	6.828	-0.486	0.5090
hsa-miR-623-001555	31.979	31.774	14.220	34.498	34.551	36.070	30.528	30.208	12.868	8.975	14.220	13.221	12.394	14.825	9.373	7.009	1.421	0.5200
hsa-miR-618-001593	23.269	21.095	1.239	22.887	23.724	19.773	22.480	26.089	4.158	-1.704	1.239	1.610	1.567	-1.472	1.325	2.890	0.248	0.8752
hsa-miR-601-001558	33.833	36.410	9.474	35.420	32.292	31.762	33.104	37.074	14.722	13.611	9.474	14.143	10.135	10.517	11.949	13.875	1.368	0.3895
hsa-miR-598-001988	29.029	33.083	13.766	32.027	33.500	32.468	29.762	40	9.918	10.284	13.766	10.750	11.343	11.223	8.607	16.801	-0.814	0.6932

hsa-miR-597-001551	30.275	28.824	7.377	28.322	30.537	29.390	30.206	31.867	11.164	6.025	7.377	7.045	8.380	8.145	9.051	8.668	-0.658	0.6025
hsa-miR-590-5p-001984	27.241	31.676	8.931	28.938	29.146	29.109	27.98	30.654	8.130	8.877	8.931	7.661	6.989	7.864	6.825	7.455	1.117	0.0299
hsa-miR-579-002398	30.08	40	10.845	30.237	32.958	30.703	32.455	31.521	10.969	17.201	10.845	8.960	10.801	9.458	11.300	8.322	2.023	0.3534
hsa-miR-574-3p-002349	22.554	28.518	5.328	26.171	26.269	26.573	26.263	27.807	3.443	5.719	5.328	4.894	4.112	5.328	5.108	4.608	0.057	0.9239
hsa-miR-573-001615	32.893	40	9.933	34.622	40	31.029	32.165	32.441	13.782	17.201	9.933	13.345	17.843	9.784	11.010	9.242	1.596	0.5465
hsa-miR-572-001614	30.725	29.111	7.917	32.069	31.774	27.876	31.43	33.306	11.614	6.312	7.917	10.792	9.617	6.631	10.275	10.107	0.001	0.9993
hsa-miR-571-001613	29.26	29.328	7.596	40	31.723	31.473	30.417	40	10.149	6.529	7.596	18.723	9.566	10.228	9.262	16.801	-0.715	0.8365
hsa-miR-550-001544	34.146	34.419	11.423	35.607	40	34.158	34.907	40	15.035	11.620	11.423	14.330	17.843	12.913	13.752	16.801	-2.225	0.1918
hsa-miR-548a-001538	32.455	40	11.130	33.683	33.368	40	34.447	32.937	13.344	17.201	11.130	12.406	11.211	18.755	13.292	9.738	0.271	0.9131
hsa-miR-543-002376	28.749	33.241	11.937	31.281	33.426	30.612	31.116	32.75	9.638	10.442	11.937	10.004	11.269	9.367	9.961	9.551	0.468	0.5069
hsa-miR-532-3p-002355	26.449	29.978	7.465	29.301	28.559	28.115	27.939	30.883	7.338	7.179	7.465	8.024	6.402	6.870	6.784	7.684	0.567	0.1397
hsa-miR-532-001518	25.944	30.379	7.057	26.967	27.677	27.142	27.349	31.1	6.833	7.580	7.057	5.690	5.520	5.897	6.194	7.901	0.412	0.5570
hsa-miR-523-002386	30.063	32.144	6.596	32.632	30.153	29.633	28.025	29.966	10.952	9.345	6.596	11.355	7.996	8.388	6.870	6.767	2.057	0.1523
hsa-miR-520e-001119	30.471	32.55	9.331	30.126	30.834	31.78	30.293	40	11.360	9.751	9.331	8.849	8.677	10.535	9.138	16.801	-1.465	0.5012
hsa-miR-520d-5p-002393	33.019	32.443	9.085	31.998	40	30.946	33.63	30.493	13.908	9.644	9.085	10.721	17.843	9.701	12.475	7.294	-0.989	0.7125
hsa-miR-520D-3P-002743	31.763	30.443	8.587	32.579	32.619	32.095	31.099	27.694	12.652	7.644	8.587	11.302	10.462	10.850	9.944	4.495	1.108	0.5807
hsa-miR-520c-3p-002400	29.745	30.282	8.481	31.273	27.439	28.275	26.603	27.17	10.634	7.483	8.481	9.996	5.282	7.030	5.448	3.971	3.716	0.0082
hsa-miR-520b-001116	32.665	30.616	10.753	32.263	31.144	33.076	31.686	33.991	13.554	7.817	10.753	10.986	8.987	11.831	10.531	10.792	0.242	0.8617
hsa-miR-519b-3p-002384	37.168	34.104	11.756	37.318	31.275	26.739	35.333	27.36	18.057	11.305	11.756	16.041	9.118	5.494	14.178	4.161	6.052	0.0764
hsa-miR-518f-002388	26.995	28.93	7.236	29.556	27.582	29.884	28.206	25.645	7.884	6.131	7.236	8.279	5.425	8.639	7.051	2.446	1.492	0.3513
hsa-miR-518d-001159	28.886	28.401	6.134	29.999	29.076	28.181	28.88	28.523	9.775	5.602	6.134	8.722	6.919	6.936	7.725	5.324	0.832	0.4966

hsa-miR-516-3p-001149	32.799	34.284	10.170	33.944	36.71	36.654	34.931	31.079	13.688	11.485	10.170	12.667	14.553	15.409	13.776	7.880	-0.902	0.6537
hsa-miR-505#-002087	27.916	32.326	8.587	30.99	32.492	31.416	31.024	34.012	8.805	9.527	8.587	9.713	10.335	10.171	9.869	10.813	-1.139	0.0171
hsa-miR-502-3p-002083	31.272	40	14.291	33.5	33.26	33.521	31.883	34.586	12.161	17.201	14.291	12.223	11.103	12.276	10.728	11.387	2.596	0.1133
hsa-miR-500-002428	28.88	33.069	17.179	28.933	33.352	28.93	28.881	33.8	9.769	10.270	17.179	7.656	11.195	7.685	7.726	10.601	1.917	0.4434
hsa-miR-497-001043	32.672	32.906	14.734	31.847	36.36	35.577	32.789	40	13.561	10.107	14.734	10.570	14.203	14.332	11.634	16.801	-2.000	0.2435
hsa-miR-486-3p-002093	26.973	28.194	7.674	27.168	28.174	26.974	27.483	32.191	7.862	5.395	7.674	5.891	6.017	5.729	6.328	8.992	-0.061	0.9523
hsa-miR-486-001278	21.772	23.528	1.100	21.65	23.133	22.02	21.873	24.337	2.661	0.729	1.100	0.373	0.976	0.775	0.718	1.138	0.314	0.5812
hsa-miR-485-3p-001277	26.585	33.504	10.642	31.578	30.957	29.886	30.017	31.815	7.474	10.705	10.642	10.301	8.800	8.641	8.862	8.616	1.051	0.2678
* hsa-miR-484-001821	19.111	22.799	0.000	21.277	22.157	21.245	21.155	23.199	0.000	0.000	0.000	0.000	0.000	0.000	0.000	0.000	NA	NA
hsa-miR-483-5p-002338	28.185	29.329	8.001	29.738	29.263	29.019	30.631	29.72	9.074	6.530	8.001	8.461	7.106	7.774	9.476	6.521	0.297	0.7354
hsa-miR-483-3p-002339	28.581	27.586	5.583	27.734	28.442	29.137	29.53	40	9.470	4.787	5.583	6.457	6.285	7.892	8.375	16.801	-3.264	0.2725
hsa-miR-454-002323	25.783	30.969	8.469	28.83	28.676	27.883	27.715	30.781	6.672	8.170	8.469	7.553	6.519	6.638	6.560	7.582	0.891	0.1158
hsa-miR-450b-5p-002207	28.719	32.788	7.177	29.987	36.052	36.269	34.325	14.392	9.608	9.989	7.177	8.710	13.895	15.024	13.170	-8.807	0.550	0.9297
hsa-miR-432-001026	26.577	32.583	13.811	30.489	30.534	31.26	29.11	33.747	7.466	9.784	13.811	9.212	8.377	10.015	7.955	10.548	0.844	0.5972
hsa-miR-425#-002302	24.862	32.658	11.972	35.392	27.966	28.006	29.395	31.264	5.751	9.859	11.972	14.115	5.809	6.761	8.240	8.065	3.205	0.1699
hsa-miR-425-5p-001516	21.319	25.194	2.457	23.445	23.8	23.048	23.404	25.461	2.208	2.395	2.457	2.168	1.643	1.803	2.249	2.262	0.318	0.1362
hsa-miR-423-5p-002340	25.798	28.641	7.167	27.648	29.706	28.839	28.912	29.116	6.687	5.842	7.167	6.371	7.549	7.594	7.757	5.917	-0.688	0.2367
hsa-miR-409-3p-002332	23.874	30.637	9.253	29.525	28.74	28.031	28.629	29.372	4.763	7.838	9.253	8.248	6.583	6.786	7.474	6.173	0.771	0.4916
hsa-miR-383-000573	33.158	34.375	10.241	32.286	35.062	40	40	36.239	14.047	11.576	10.241	11.009	12.905	18.755	18.845	13.040	-4.168	0.0846
hsa-miR-382-000572	23.912	31.34	10.130	32.835	29.681	30.865	29.693	33.859	4.801	8.541	10.130	11.558	7.524	9.620	8.538	10.660	-0.328	0.8476
hsa-miR-378-002243	28.96	29.985	7.759	30.247	29.44	28.217	28.553	29.92	9.849	7.186	7.759	8.970	7.283	6.972	7.398	6.721	1.347	0.1070

hsa-miR-378-000567	33.421	31.604	12.686	39.216	33.6	34.329	40	34.425	14.310	8.805	12.686	17.939	11.443	13.084	18.845	11.226	-0.215	0.9369
hsa-miR-376c-002122	25.658	34.115	8.545	30.762	29.748	30.179	29.746	31.858	6.547	11.316	8.545	9.485	7.591	8.934	8.591	8.659	0.530	0.6393
hsa-miR-376a-000565	26.552	34.967	10.241	32.525	30.34	29.465	29.97	33.22	7.441	12.168	10.241	11.248	8.183	8.220	8.815	10.021	1.465	0.2568
hsa-miR-375-000564	26.418	28.135	5.614	26.926	27.533	26.496	27.197	27.535	7.307	5.336	5.614	5.649	5.376	5.251	6.042	4.336	0.725	0.2530
hsa-miR-374-000563	24.063	31.201	6.894	27.999	27.094	27.225	26.837	32.303	4.952	8.402	6.894	6.722	4.937	5.980	5.682	9.104	0.317	0.7944
hsa-miR-370-002275	24.628	31.669	12.658	28.656	30.828	27.787	29.549	30	5.517	8.870	12.658	7.379	8.671	6.542	8.394	6.801	1.004	0.5686
hsa-miR-365-001020	29.392	31.589	8.042	29.89	30.096	30.597	29.908	33.372	10.281	8.790	8.042	8.613	7.939	9.352	8.753	10.173	-0.123	0.8609
hsa-miR-363-001271	27.277	32.26	8.030	28.665	29.581	27.828	29.588	37.109	8.166	9.461	8.030	7.388	7.424	6.583	8.433	13.910	-0.826	0.6578
hsa-miR-34b-002102	27.853	29.648	11.387	30.048	40	30.997	28.176	30.426	8.742	6.849	11.387	8.771	17.843	9.752	7.021	7.227	-1.523	0.6047
hsa-miR-34a-000426	26.29	27.879	2.875	27.336	26.481	25.514	25.441	28.314	7.179	5.080	2.875	6.059	4.324	4.269	4.286	5.115	0.800	0.4509
hsa-miR-346-000553	28.505	26.328	4.697	27.101	28.842	26.764	26.907	28.36	9.394	3.529	4.697	5.824	6.685	5.519	5.752	5.161	0.082	0.9537
hsa-miR-345-002186	25.521	30.117	6.862	27.93	28.517	28.463	28.108	28.953	6.410	7.318	6.862	6.653	6.360	7.218	6.953	5.754	0.240	0.5557
hsa-miR-342-3p-002260	21.193	25.729	2.460	24.592	24.469	24.848	24.742	26.633	2.082	2.930	2.460	3.315	2.312	3.603	3.587	3.434	-0.537	0.2393
hsa-miR-340-002258	26.966	40	8.546	30.777	31.251	32.041	29.926	40	7.855	17.201	8.546	9.500	9.094	10.796	8.771	16.801	-0.590	0.8435
hsa-miR-33a-002135	26.524	25.335	3.470	40	27.806	26.255	27.104	27.257	7.413	2.536	3.470	18.723	5.649	5.010	5.949	4.058	2.869	0.4975
hsa-miR-339-5p-002257	23.616	34.696	8.382	29.083	30.189	31.961	32.12	34.914	4.505	11.897	8.382	7.806	8.032	10.716	10.965	11.715	-2.210	0.2588
hsa-miR-339-3p-002184	25.315	32.046	8.059	29.356	29.069	29.226	30.117	30.704	6.204	9.247	8.059	8.079	6.912	7.981	8.962	7.505	0.057	0.9429
hsa-miR-338-5P-002658	33.84	34.224	11.844	33.291	33.332	34.695	32.951	33.178	14.729	11.425	11.844	12.014	11.175	13.450	11.796	9.979	0.903	0.4200
hsa-miR-335#-002185	25.2	34.31	10.415	40	29.656	29.465	29.263	31.613	6.089	11.511	10.415	18.723	7.499	8.220	8.108	8.414	3.624	0.2609
hsa-miR-335-000546	24.919	30.986	7.706	30.205	28.628	28.791	29.263	32.791	5.808	8.187	7.706	8.928	6.471	7.546	8.108	9.592	-0.272	0.7799
hsa-miR-331-000545	22.56	27.768	4.491	26.413	26.336	26.429	26.24	28.959	3.449	4.969	4.491	5.136	4.179	5.184	5.085	5.760	-0.541	0.3227

hsa-miR-330-000544	26.411	28.052	9.373	31.756	30.486	29.369	29.902	40	7.300	5.253	9.373	10.479	8.329	8.124	8.747	16.801	-2.399	0.3667
hsa-miR-328-000543	22.18	27.262	5.162	26.068	26.065	25.822	26.819	26.957	3.069	4.463	5.162	4.791	3.908	4.577	5.664	3.758	-0.106	0.8726
hsa-miR-324-5p-000539	25.166	32.8	7.222	28.019	28.918	31.342	28.822	31.829	6.055	10.001	7.222	6.742	6.761	10.097	7.667	8.630	-0.784	0.5118
hsa-miR-324-3p-002161	26.248	30.002	8.189	28.924	29.085	27.97	28.313	29.212	7.137	7.203	8.189	7.647	6.928	6.725	7.158	6.013	0.838	0.0521
hsa-miR-323-3p-002227	29.15	32.317	10.089	32.843	32.106	32.648	31.714	34.9	10.039	9.518	10.089	11.566	9.949	11.403	10.559	11.701	-0.600	0.3522
hsa-miR-320B-002844	26.172	28.797	6.319	28.359	28.767	26.546	27.686	30.503	7.061	5.998	6.319	7.082	6.610	5.301	6.531	7.304	0.179	0.7336
hsa-miR-320-002277	20.811	24.326	1.712	22.885	23.373	22.426	22.382	23.443	1.700	1.527	1.712	1.608	1.216	1.181	1.227	0.244	0.670	0.0670
hsa-miR-31-002279	28.704	40	7.223	40	30.129	30.994	29.112	35.76	9.593	17.201	7.223	18.723	7.972	9.749	7.957	12.561	3.625	0.2981
hsa-miR-30e-3p-000422	25.369	34.079	7.125	29.038	28.482	28.987	29.178	29.485	6.258	11.280	7.125	7.761	6.325	7.742	8.023	6.286	1.012	0.4442
hsa-miR-30d-000420	24.743	29.153	5.362	27.299	27.184	26.167	27.091	30.114	5.632	6.354	5.362	6.022	5.027	4.922	5.936	6.915	0.143	0.7942
hsa-miR-30c-000419	21.231	27.14	3.185	25.156	24.829	24.84	24.411	27.582	2.120	4.341	3.185	3.879	2.672	3.595	3.256	4.383	-0.095	0.8796
hsa-miR-30b-000602	21.563	27.551	3.489	25.316	25.321	25.255	24.886	27.939	2.452	4.752	3.489	4.039	3.164	4.010	3.731	4.740	-0.228	0.7118
hsa-miR-30a-5p-000417	22.264	26.514	3.238	25.164	25.157	24.343	24.454	27.231	3.153	3.715	3.238	3.887	3.000	3.098	3.299	4.032	0.141	0.6501
hsa-miR-30a-3p-000416	27.303	40	5.352	28.458	27.792	29.247	28.577	31.909	8.192	17.201	5.352	7.181	5.635	8.002	7.422	8.710	2.039	0.5024
hsa-miR-302c-000533	32.242	33.719	6.350	32.657	35.961	35.061	35.681	37.124	13.131	10.920	6.350	11.380	13.804	13.816	14.526	13.925	-3.572	0.0890
hsa-miR-302b-000531	31.424	36.935	14.674	36.183	40	33.754	37.048	39.137	12.313	14.136	14.674	14.906	17.843	12.509	15.893	15.938	-1.539	0.2799
hsa-miR-302a-000529	28.149	28.5	7.222	31.025	30.731	29.68	30.174	32.33	9.038	5.701	7.222	9.748	8.574	8.435	9.019	9.131	-0.863	0.4174
hsa-miR-301-000528	25.868	30.755	7.839	28.823	29.103	28.404	27.763	32.517	6.757	7.956	7.839	7.546	6.946	7.159	6.608	9.318	0.017	0.9814
hsa-miR-29c-000587	26.983	30.562	7.973	29.325	29.148	29.49	29.068	32.195	7.872	7.763	7.973	8.048	6.991	8.245	7.913	8.996	-0.122	0.7893
hsa-miR-29a-002112	24.913	29.714	5.734	27.892	27.4	27.822	27.65	30.431	5.802	6.915	5.734	6.615	5.243	6.577	6.495	7.232	-0.120	0.8218
hsa-miR-296-000527	26.337	28.391	6.136	26.32	27.952	27.821	26.421	31.286	7.226	5.592	6.136	5.043	5.795	6.576	5.266	8.087	-0.432	0.5971

hsa-miR-28-3p-002446	23.861	30.594	6.600	28.222	28.087	28.154	28.185	28.989	4.750	7.795	6.600	6.945	5.930	6.909	7.030	5.790	0.108	0.8874
hsa-miR-28-000411	25.991	40	12.521	30.268	30.437	31.1	32.756	33.994	6.880	17.201	12.521	8.991	8.280	9.855	11.601	10.795	1.265	0.6243
hsa-miR-27b-000409	25.984	31.487	8.793	31.109	29.233	28.968	29.597	31.209	6.873	8.688	8.793	9.832	7.076	7.723	8.442	8.010	0.734	0.3369
hsa-miR-27a-000408	25.097	29.787	7.197	28.879	28.736	28.547	28.145	31.784	5.986	6.988	7.197	7.602	6.579	7.302	6.990	8.585	-0.421	0.4769
hsa-miR-26b-000407	23.779	27.964	4.675	26.559	25.98	25.617	25.469	29.536	4.668	5.165	4.675	5.282	3.823	4.372	4.314	6.337	0.236	0.7069
hsa-miR-26a-000405	20.868	28.074	3.422	25.304	24.738	25.149	24.789	27.594	1.757	5.275	3.422	4.027	2.581	3.904	3.634	4.395	-0.008	0.9924
hsa-miR-25-000403	23.724	26.862	3.726	24.575	25.355	24.881	24.874	28.359	4.613	4.063	3.726	3.298	3.198	3.636	3.719	5.160	-0.003	0.9951
hsa-miR-24-000402	18.742	25.008	0.994	23.26	22.538	22.785	22.628	25.592	-0.369	2.209	0.994	1.983	0.381	1.540	1.473	2.393	-0.242	0.7481
hsa-miR-224-002099	24.922	40	6.973	29.112	28.013	27.838	32.37	33.976	5.811	17.201	6.973	7.835	5.856	6.593	11.215	10.777	0.845	0.7879
hsa-miR-223#-002098	24.765	31.784	7.034	29.445	29.541	28.55	27.33	31.366	5.654	8.985	7.034	8.168	7.384	7.305	6.175	8.167	0.203	0.8177
hsa-miR-223-002295	14.463	21.929	-2.333	19.697	18.886	19.117	19.294	21.102	-4.648	-0.870	-2.333	-1.580	-3.271	-2.128	-1.861	-2.097	-0.018	0.9842
hsa-miR-222-002276	21.144	26.758	3.583	25.241	24.476	24.356	24.725	26.131	2.033	3.959	3.583	3.964	2.319	3.111	3.570	2.932	0.402	0.4822
hsa-miR-221-000524	20.896	29.517	8.660	28.998	25.514	26.512	26.905	27.07	1.785	6.718	8.660	7.721	3.357	5.267	5.750	3.871	1.660	0.3694
hsa-miR-22#-002301	25.979	30.377	12.841	31.605	30.687	29.835	30.196	40	6.868	7.578	12.841	10.328	8.530	8.590	9.041	16.801	-1.337	0.6065
hsa-miR-22-000398	26.062	33.602	8.720	27.756	32.719	28.145	30.056	32.936	6.951	10.803	8.720	6.479	10.562	6.900	8.901	9.737	-0.787	0.5555
hsa-miR-215-000518	28.001	28.878	6.464	27.759	28.607	28.422	27.337	31.872	8.890	6.079	6.464	6.482	6.450	7.177	6.182	8.673	-0.142	0.8734
hsa-miR-214-002306	29.756	31.609	10.788	30.708	31.222	29.444	30.899	34.001	10.645	8.810	10.788	9.431	9.065	8.199	9.744	10.802	0.466	0.5467
hsa-miR-212-000515	33.97	30.722	9.661	32.195	29.612	30.568	30.295	30.265	14.859	7.923	9.661	10.918	7.455	9.323	9.140	7.066	2.594	0.1783
hsa-miR-210-000512	24.373	27.758	6.005	24.953	26.185	25.07	25.129	26.15	5.262	4.959	6.005	3.676	4.028	3.825	3.974	2.951	1.281	0.0720
hsa-miR-21-000397	21.965	26.038	2.696	24.533	24.385	24.072	24.181	27.501	2.854	3.239	2.696	3.256	2.228	2.827	3.026	4.302	-0.084	0.8636
hsa-miR-20b-001014	23.204	26.91	3.845	25.085	27.223	26.744	28.101	29.991	4.093	4.111	3.845	3.808	5.066	5.499	6.946	6.792	-2.112	0.0187
hsa-miR-20a-000580	19.847	23.453	0.479	21.724	22.074	21.767	21.178	24.428	0.736	0.654	0.479	0.447	-0.083	0.522	0.023	1.229	0.156	0.6433

hsa-miR-208-000511	28.807	27.438	13.551	26.278	28.882	28.573	27.934	34.002	9.696	4.639	13.551	5.001	6.725	7.328	6.779	10.803	0.313	0.8993
hsa-miR-204-000508	29.129	32.054	13.506	29.705	30.456	30.902	31.183	31.755	10.018	9.255	13.506	8.428	8.299	9.657	10.028	8.556	1.167	0.3855
hsa-miR-203-000507	27.855	31.138	13.596	32.495	32.484	33.851	40	32.882	8.744	8.339	13.596	11.218	10.327	12.606	18.845	9.683	-2.391	0.3699
hsa-miR-202-002363	33.533	30.319	12.755	31.952	34.346	32.893	31.932	32.669	14.422	7.520	12.755	10.675	12.189	11.648	10.777	9.470	0.322	0.8505
hsa-miR-200c-002300	25.673	30.432	12.297	29.832	28.664	31.612	33.694	31.464	6.562	7.633	12.297	8.555	6.507	10.367	12.539	8.265	-0.658	0.7281
hsa-miR-200b-002251	27.361	29.609	12.690	32.211	28.806	31.022	28.778	30.434	8.250	6.810	12.690	10.934	6.649	9.777	7.623	7.235	1.850	0.2741
hsa-miR-19b-1#-002425	29.068	31.63	10.172	32.614	32.014	30.452	33.833	34.002	9.957	8.831	10.172	11.337	9.857	9.207	12.678	10.803	-0.562	0.5638
hsa-miR-19b-000396	18.229	21.072	-1.470	19.433	20.297	18.806	19.237	21.447	-0.882	-1.727	-1.470	-1.844	-1.860	-2.439	-1.918	-1.752	0.511	0.1052
hsa-miR-19a-000395	23.227	26.067	3.674	24.396	24.989	24.094	23.848	26.819	4.116	3.268	3.674	3.119	2.832	2.849	2.693	3.620	0.546	0.1259
hsa-miR-199a-3p-002304	22.9	30.067	6.674	28.093	26.289	27.286	27.301	30.44	3.789	7.268	6.674	6.816	4.132	6.041	6.146	7.241	0.247	0.8177
hsa-miR-199a-000498	27.844	40	7.963	31.258	40	30.49	30.438	35.053	8.733	17.201	7.963	9.981	17.843	9.245	9.283	11.854	-1.087	0.7234
hsa-miR-197-000497	22.932	27.966	3.861	25.458	26.316	26.097	26.385	27.62	3.821	5.167	3.861	4.181	4.159	4.852	5.230	4.421	-0.408	0.3417
hsa-miR-195-000494	24.04	27.273	3.886	25.096	25.867	24.977	24.777	29.076	4.929	4.474	3.886	3.819	3.710	3.732	3.622	5.877	0.042	0.9484
hsa-miR-194-000493	26.643	30.524	5.808	25.448	28.596	27.365	27.964	32.677	7.532	7.725	5.808	4.171	6.439	6.120	6.809	9.478	-0.903	0.4563
hsa-miR-193b-002367	27.662	28.767	7.222	28.481	28.27	28.555	28.195	29.517	8.551	5.968	7.222	7.204	6.113	7.310	7.040	6.318	0.541	0.4115
hsa-miR-193a-5p-002281	27.407	29.306	6.250	28.252	29.727	28.179	30.158	29.445	8.296	6.507	6.250	6.975	7.570	6.934	9.003	6.246	-0.431	0.5840
hsa-miR-193a-3p-002250	33.543	34.727	14.436	34.011	40	35.897	40	33.84	14.432	11.928	14.436	12.734	17.843	14.652	18.845	10.641	-2.113	0.3449
hsa-miR-192-000491	26.938	29.587	5.894	27.279	27.304	26.846	27.019	29.913	7.827	6.788	5.894	6.002	5.147	5.601	5.864	6.714	0.796	0.2054
hsa-miR-191-002299	18.547	25.399	1.545	23.266	22.934	22.786	22.601	24.75	-0.564	2.600	1.545	1.989	0.777	1.541	1.446	1.551	0.064	0.9335
hsa-miR-190b-002263	30.479	36.626	13.319	29.824	32.916	30.464	28.874	28.749	11.368	13.827	13.319	8.547	10.759	9.219	7.719	5.550	3.453	0.0790
hsa-miR-18b-002217	26.825	33.208	12.857	29.041	30.771	29.32	29.229	40	7.714	10.409	12.857	7.764	8.614	8.075	8.074	16.801	-0.705	0.7871

hsa-miR-18a#-002423	31.843	40	9.946	34.231	40	32.573	32.771	32.51	12.732	17.201	9.946	12.954	17.843	11.328	11.616	9.311	0.684	0.7836
hsa-miR-18a-002422	24.586	28.926	5.809	26.605	27.995	26.572	26.13	32.085	5.475	6.127	5.809	5.328	5.838	5.327	4.975	8.886	-0.572	0.5720
hsa-miR-186-002285	22.927	28.435	5.135	26.433	26.297	25.992	25.531	27.993	3.816	5.636	5.135	5.156	4.140	4.747	4.376	4.794	0.421	0.3740
hsa-miR-185-002271	24.955	28.082	5.226	26.282	23.714	24.932	23.093	25.653	5.844	5.283	5.226	5.005	1.557	3.687	1.938	2.454	2.931	0.0046
hsa-miR-183-002269	30.698	33.942	14.904	30.766	35.27	30.165	33.801	40	11.587	11.143	14.904	9.489	13.113	8.920	12.646	16.801	-1.089	0.6027
hsa-miR-1825-002907	25.731	27.256	2.953	28.55	27.357	28.451	25.278	26.589	6.620	4.457	2.953	7.273	5.200	7.206	4.123	3.390	0.346	0.7986
hsa-miR-181c-000482	28.73	40	12.255	32.787	33.37	33.487	33.138	37.782	9.619	17.201	12.255	11.510	11.213	12.242	11.983	14.583	0.141	0.9403
hsa-miR-181a-000480	24.198	33.284	6.056	28.07	28.232	27.964	28.228	30.995	5.087	10.485	6.056	6.793	6.075	6.719	7.073	7.796	0.189	0.8862
hsa-miR-17-002308	19.294	22.704	0.124	21.1	21.664	20.804	20.618	23.784	0.183	-0.095	0.124	-0.177	-0.493	-0.441	-0.537	0.585	0.230	0.4663
hsa-miR-16-000391	18.728	22.256	-0.969	20.507	20.433	19.747	19.505	23.506	-0.383	-0.543	-0.969	-0.770	-1.724	-1.498	-1.650	0.307	0.475	0.4059
hsa-miR-15b#-002173	24.647	33.452	9.572	28.454	27.795	27.954	30.081	40	5.536	10.653	9.572	7.177	5.638	6.709	8.926	16.801	-1.284	0.6664
hsa-miR-15b-000390	23.316	29.865	6.045	27.415	26.957	27.205	26.968	31.935	4.205	7.066	6.045	6.138	4.800	5.960	5.813	8.736	-0.464	0.6712
hsa-miR-15a#-002419	30.664	30.697	13.000	31.942	31.567	31.474	29.821	40	11.553	7.898	13.000	10.665	9.410	10.229	8.666	16.801	-0.497	0.8270
hsa-miR-15a-000389	25.3	30.718	7.883	26.833	29.163	29.966	29.57	29.368	6.189	7.919	7.883	5.556	7.006	8.721	8.415	6.169	-0.691	0.4464
hsa-miR-155-002623	26.835	31.729	8.520	30.202	31.187	30.189	31.07	30.93	7.724	8.930	8.520	8.925	9.030	8.944	9.915	7.731	-0.380	0.5053
hsa-miR-151-5P-002642	25.079	30.993	7.897	29.2	28.208	27.507	28.821	31.925	5.968	8.194	7.897	7.923	6.051	6.262	7.666	8.726	0.319	0.7084
hsa-miR-151-3p-002254	22.348	29.38	5.149	26.985	26.436	26.77	26.463	27.839	3.237	6.581	5.149	5.708	4.279	5.525	5.308	4.640	0.231	0.7780
hsa-miR-150-000473	19.16	23.175	0.208	22.176	22.112	22.678	22.861	25.057	0.049	0.376	0.208	0.899	-0.045	1.433	1.706	1.858	-0.855	0.1449
hsa-miR-148b-000471	28.763	33.337	13.429	32.534	31.808	31.95	32.076	40	9.652	10.538	13.429	11.257	9.651	10.705	10.921	16.801	-0.800	0.6788
hsa-miR-148a-000470	27.057	32.293	7.801	28.848	29.145	29.654	29.224	32.223	7.946	9.494	7.801	7.571	6.988	8.409	8.069	9.024	0.080	0.8995
hsa-miR-146b-001097	22.654	27.715	5.406	27.142	26.068	26.857	26.729	29.296	3.543	4.916	5.406	5.865	3.911	5.612	5.574	6.097	-0.366	0.6162

hsa-miR-146a-000468	18.796	25.986	2.005	24.284	23.634	23.679	23.779	25.224	-0.315	3.187	2.005	3.007	1.477	2.434	2.624	2.025	-0.169	0.8523
hsa-miR-145-002278	24.088	30.623	5.985	27.476	28.143	27.908	28.016	29.748	4.977	7.824	5.985	6.199	5.986	6.663	6.861	6.549	-0.269	0.6890
hsa-miR-144#-002148	27.922	30.178	7.240	28.62	28.698	29.181	29.306	32.629	8.811	7.379	7.240	7.343	6.541	7.936	8.151	9.430	-0.321	0.6651
hsa-miR-144-002676	29.39	30.456	7.916	30.387	30.064	29.354	27.813	32.804	10.279	7.657	7.916	9.110	7.907	8.109	6.658	9.605	0.671	0.4618
hsa-miR-143-002249	28.008	40	14.179	31.02	30.787	29.378	30.234	32.502	8.897	17.201	14.179	9.743	8.630	8.133	9.079	9.303	3.719	0.1515
hsa-miR-142-5p-002248	26.917	31.878	7.484	32.672	31.106	30.726	30.352	40	7.806	9.079	7.484	11.395	8.949	9.481	9.197	16.801	-2.166	0.3571
hsa-miR-142-3p-000464	23.856	28.689	4.425	26.467	25.449	25.726	26.193	28.829	4.745	5.890	4.425	5.190	3.292	4.481	5.038	5.630	0.452	0.4779
hsa-miR-140-3p-002234	27.207	31.157	7.661	29.301	29.381	28.27	28.053	31.769	8.096	8.358	7.661	8.024	7.224	7.025	6.898	8.570	0.606	0.2188
hsa-miR-139-5p-002289	22.809	29.511	5.233	27.145	26.08	26.419	26.676	29.14	3.698	6.712	5.233	5.868	3.923	5.174	5.521	5.941	0.238	0.7693
hsa-miR-139-3p-002313	24.453	28.285	13.298	27.912	27.785	28.457	27.064	30.589	5.342	5.486	13.298	6.635	5.628	7.212	5.909	7.390	1.155	0.5901
hsa-miR-133b-002247	28.921	31.879	17.179	32.63	34.737	31.699	31.45	31.187	9.810	9.080	17.179	11.353	12.580	10.454	10.295	7.988	1.526	0.4963
hsa-miR-133a-002246	25.827	31.421	7.113	28.968	28.848	28.543	28.739	30.882	6.716	8.622	7.113	7.691	6.691	7.298	7.584	7.683	0.221	0.6592
hsa-miR-132-000457	25.583	30.565	7.899	27.574	28.904	28.311	28.147	29.824	6.472	7.766	7.899	6.297	6.747	7.066	6.992	6.625	0.251	0.5985
hsa-miR-130b-000456	24.93	30.269	8.274	28.096	27.822	27.815	27.712	29.728	5.819	7.470	8.274	6.819	5.665	6.570	6.557	6.529	0.765	0.2458
hsa-miR-130a-000454	24.515	29.418	5.930	28.923	28.278	27.817	27.903	30.071	5.404	6.619	5.930	7.646	6.121	6.572	6.748	6.872	-0.178	0.7461
hsa-miR-1305-002867	31.504	33.239	10.019	32.608	33.339	31.1	30.818	30.471	12.393	10.440	10.019	11.331	11.182	9.855	9.663	7.272	1.553	0.1684
hsa-miR-1298-002861	27.161	24.594	3.988	27.258	25.073	25.652	28.348	24.485	8.050	1.795	3.988	5.981	2.916	4.407	7.193	1.286	1.003	0.6047
hsa-miR-1290-002863	28.415	31.914	7.837	29.561	30.3	31.189	30.781	30.061	9.304	9.115	7.837	8.284	8.143	9.944	9.626	6.862	-0.009	0.9918
hsa-miR-128a-002216	25.806	40	7.530	29.333	31.463	29.979	29.556	31.789	6.695	17.201	7.530	8.056	9.306	8.734	8.401	8.590	1.113	0.6822
hsa-miR-1285-002822	28.931	33.871	11.176	33.414	40	31.983	40	34.645	9.820	11.072	11.176	12.137	17.843	10.738	18.845	11.446	-3.667	0.1799
hsa-miR-1276-002843	34.99	31.811	11.192	40	35.991	32.096	34.703	40	15.879	9.012	11.192	18.723	13.834	10.851	13.548	16.801	-0.057	0.9829

hsa-miR-1274B-002884	21.082	22.155	-0.533	21.926	22.909	21.935	21.843	22.608	1.971	-0.644	-0.533	0.649	0.752	0.690	0.688	-0.591	-0.024	0.9738
hsa-miR-1274A-002883	24.967	26.565	4.073	26.531	27.214	26.542	26.461	27.811	5.856	3.766	4.073	5.254	5.057	5.297	5.306	4.612	-0.331	0.5609
hsa-miR-127-000452	26.279	30.983	13.012	29.139	29.359	29.811	31.361	30.297	7.168	8.184	13.012	7.862	7.202	8.566	10.206	7.098	0.788	0.6279
hsa-miR-1262-002852	32.516	30.191	9.722	33.239	31.173	30.626	27.758	30.371	13.405	7.392	9.722	11.962	9.016	9.381	6.603	7.172	2.577	0.1490
hsa-miR-1260-002896	26.791	27.026	3.890	28.052	28.239	26.791	27.394	28.841	7.680	4.227	3.890	6.775	6.082	5.546	6.239	5.642	-0.234	0.8203
hsa-miR-126#-000451	22.456	27.971	3.864	26.312	25.186	25.621	25.206	28.78	3.345	5.172	3.864	5.035	3.029	4.376	4.051	5.581	0.095	0.8953
hsa-miR-126-002228	19.052	25.299	1.182	23.589	22.395	22.775	22.44	25.681	-0.059	2.500	1.182	2.312	0.238	1.530	1.285	2.482	0.100	0.8985
hsa-miR-125b-000449	27.629	30.227	7.815	29.267	29.201	29.187	28.913	31.027	8.518	7.428	7.815	7.990	7.044	7.942	7.758	7.828	0.295	0.3703
hsa-miR-125a-5p-002198	27.68	35.357	10.467	33.714	31.183	32.196	34.383	40	8.569	12.558	10.467	12.437	9.026	10.951	13.228	16.801	-1.494	0.4733
hsa-miR-1255B-002801	29.497	40	11.248	31.65	32.4	31.062	30.599	32.616	10.386	17.201	11.248	10.373	10.243	9.817	9.444	9.417	2.572	0.2161
hsa-miR-1247-002893	29.491	29.144	9.299	40	30.443	28.366	30.819	31.943	10.380	6.345	9.299	18.723	8.286	7.121	9.664	8.744	2.733	0.3818
hsa-miR-1233-002768	26.317	27.315	5.729	28.68	27.234	27.91	26.856	27.136	7.206	4.516	5.729	7.403	5.077	6.665	5.701	3.937	0.869	0.3662
hsa-miR-1227-002769	30.517	40	10.817	33.386	33.307	31.17	32.298	31.799	11.406	17.201	10.817	12.109	11.150	9.925	11.143	8.600	2.679	0.1661
hsa-miR-1225-3P-002766	31.844	30.823	8.017	32.938	35.913	30.085	32.346	31.124	12.733	8.024	8.017	11.661	13.756	8.840	11.191	7.925	-0.319	0.8644
hsa-miR-122-002245	23.304	24.821	2.009	24.218	23.514	23.997	24.576	27.338	4.193	2.022	2.009	2.941	1.357	2.752	3.421	4.139	-0.126	0.8779
hsa-miR-1208-002880	28.137	28.464	6.179	28.754	25.566	27.92	27.84	27.027	9.026	5.665	6.179	7.477	3.409	6.675	6.685	3.828	1.937	0.1480
hsa-miR-1183-002841	27.489	27.771	5.796	29.657	29.461	26.136	27.178	23.644	8.378	4.972	5.796	8.380	7.304	4.891	6.023	0.445	2.216	0.2583
hsa-miR-1180-002847	30.671	34.144	8.274	31.491	31.106	30.256	29.591	32.647	11.560	11.345	8.274	10.214	8.949	9.011	8.436	9.448	1.387	0.1611
hsa-miR-10b#-002315	30.279	30.029	9.846	31.226	30.476	29.903	31.093	32.304	11.168	7.230	9.846	9.949	8.319	8.658	9.938	9.105	0.543	0.5784
hsa-miR-10b-002218	33.807	38.562	9.955	32.79	35.332	34.396	32.602	40	14.696	15.763	9.955	11.513	13.175	13.151	11.447	16.801	-0.662	0.7205
hsa-miR-10a-000387	28.536	40	7.960	32.548	31.726	31.653	30.876	40	9.425	17.201	7.960	11.271	9.569	10.408	9.721	16.801	-0.161	0.9540

hsa-miR-106b#-002380	22.832	28.263	9.890	28.27	32.134	27.513	30.133	29.41	3.721	5.464	9.890	6.993	9.977	6.268	8.978	6.211	-1.342	0.4422
hsa-miR-106b-000442	23.528	26.387	3.812	24.84	25.178	24.378	24.342	27.572	4.417	3.588	3.812	3.563	3.021	3.133	3.187	4.373	0.417	0.3155
hsa-miR-106a-002169	19.232	22.775	0.162	21.141	21.505	20.895	20.494	23.803	0.121	-0.024	0.162	-0.136	-0.652	-0.350	-0.661	0.604	0.295	0.3997
hsa-miR-103-000439	23.132	28.58	4.737	26.955	25.777	26.165	26.567	29.933	4.021	5.781	4.737	5.678	3.620	4.920	5.412	6.734	-0.117	0.8843
hsa-miR-101-002253	28.585	30.836	8.355	30.179	29.005	28.647	29.552	32.87	9.474	8.037	8.355	8.902	6.848	7.402	8.397	9.671	0.612	0.4236
hsa-miR-100-000437	28.003	29.854	9.555	29.6	30.171	29.146	29.96	28.177	8.892	7.055	9.555	8.323	8.014	7.901	8.805	4.978	1.032	0.3460
hsa-let-7g-002282	23.556	28.819	5.672	27.279	26.937	26.395	25.954	31.292	4.445	6.020	5.672	6.002	4.780	5.150	4.799	8.093	-0.171	0.8554
hsa-let-7e-002406	22.002	28.837	3.723	26.752	26.262	27.487	26.92	28.865	2.891	6.038	3.723	5.475	4.105	6.242	5.765	5.666	-0.913	0.3417
hsa-let-7d-002283	23.969	32.097	6.178	28.018	27.477	28.346	27.593	30.194	4.858	9.298	6.178	6.741	5.320	7.101	6.438	6.995	0.305	0.7786
hsa-let-7c-000379	28.298	32.481	10.196	29.794	30.385	31.34	30.885	31.7	9.187	9.682	10.196	8.517	8.228	10.095	9.730	8.501	0.257	0.6741
hsa-let-7b-002619	21.437	26.37	2.462	23.849	24.883	23.74	23.128	25.869	2.326	3.571	2.462	2.572	2.726	2.495	1.973	2.670	0.267	0.4583
hsa-let-7a-000377	23.376	38.207	8.699	29.308	29.516	30.51	30.325	34.65	4.265	15.408	8.699	8.031	7.359	9.265	9.170	11.451	-0.211	0.9363

*hsa-miR-484 was used as normalizer

Table S3. Expression level of miR-185-5p in the validation cohort.

	Sample	Ct values						Δ Ct values	Fold change	Average Fold Change (mean ± SE)	p-value (Mann-Whitney test)
		miR-185-5p			miR-484						
		Rep. 1	Rep. 2	Rep. 3	Rep. 1	Rep. 2	Rep. 3				
ARVC (N = 37)	63	25.6786	25.4820	25.4335	28.0547	27.9408	28.0750	-2.4922	1.8600	2.3385 ± 0.19	< 0.0001
	24	24.4685	24.3725	NA	27.2371	27.4200	NA	-2.9080	2.4815		
	15	27.3369	27.2086	NA	29.1980	NA	29.6671	-2.1598	1.4773		
	22	28.4402	NA	28.9445	31.2885	31.3244	31.5348	-2.6902	2.1337		
	27	25.6094	25.6243	25.6029	26.5216	27.5216	28.5216	-1.9094	1.2419		
	55	26.2758	26.2907	26.2693	27.1475	27.0004	NA	-0.7954	0.5738		
	3	25.7141	25.9363	25.6562	26.5282	NA	26.8051	-0.8977	0.6160		
	62	25.2524	25.2530	25.1848	27.3965	27.3808	NA	-2.1586	1.4761		
	94	24.3322	24.1609	NA	26.5168	26.5835	26.4374	-2.2660	1.5902		
	95	25.6046	26.0264	25.7754	27.9456	28.3628	NA	-2.3521	1.6879		
	102	27.4089	27.3833	27.4518	29.2309	28.7863	29.2203	-1.6645	1.0480		
	109	27.3003	27.3678	27.2314	29.2149	28.9307	NA	-1.7730	1.1299		
	107	28.7093	28.3288	28.3771	31.0752	31.2084	31.3285	-2.7323	2.1970		
	115	27.8852	27.4852	27.4252	30.9942	30.9242	31.0642	-3.3957	3.4794		
	105	28.7052	28.8652	28.7252	31.6942	31.6742	31.9542	-3.0090	2.6614		
	35	28.6852	28.6552	NA	31.6142	32.0642	32.2642	-3.3107	3.2804		
	120	24.3480	23.9412	23.6104	26.2106	25.6734	26.1839	-2.0561	1.3748		
	93	24.8406	24.9494	24.8745	28.0773	27.6380	28.0301	-3.0270	2.6948		
	100	25.9467	26.2774	26.2041	29.3027	29.1263	29.1268	-3.0425	2.7240		
	103	25.8505	22.2179	26.2626	27.9512	27.8792	28.4319	-3.3104	3.2798		
	114	26.1547	26.0098	26.0808	28.7145	28.9336	28.8623	-2.7550	2.2318		
	57	25.4724	25.5090	25.5138	28.7118	28.8371	28.4461	-3.1667	2.9687		
	5	21.6544	21.9267	22.1810	25.4925	25.0921	25.3085	-3.3770	3.4346		
	97	24.2503	24.1152	23.9950	27.9402	27.9216	27.7690	-3.7568	4.4690		
	17	29.0662	28.9478	28.7938	31.5091	32.1002	31.1299	-2.6438	2.0661		
	47	25.8757	25.6763	25.6905	29.3031	29.4486	29.3283	-3.6125	4.0437		
	7	24.4096	24.3433	NA	27.6772	27.9235	27.5462	-3.3392	3.3458		
	60	26.0847	25.9654	26.0118	27.4710	27.5502	NA	-1.4899	0.9286		
	32	25.7112	24.9626	25.5896	26.7170	26.8324	26.7504	-1.3454	0.8401		
	11	23.5951	23.5484	23.4693	25.8594	25.7973	25.8719	-2.3052	1.6340		
	46	24.0192	23.8871	24.0156	27.6134	27.5489	27.3919	-3.5441	3.8564		
	99	24.2484	24.2957	24.2929	28.0841	27.9744	28.2507	-3.8241	4.6823		
	121	27.0986	27.0386	26.9186	30.3676	NA	30.2676	-3.2990	3.2539		

	**45**	26.7186	26.8086	26.0886	NA	29.0276	29.2076	-2.5790	1.9755		
	**56**	27.8786	28.0186	27.8586	31.6476	31.6776	NA	-3.7440	4.4296		
	**92**	29.4169	29.4369	29.3369	31.8142	31.7942	NA	-2.4073	1.7538		
	**9**	29.5769	29.2169	29.3269	NA	31.5942	31.7042	-2.2757	1.6009		
	**71**	28.6860	28.7829	28.3868	29.5573	NA	29.3077	-0.8139	0.5812		
	**76**	28.7989	28.6141	NA	30.5339	29.4264	30.9678	-1.6028	1.0042		
	**69**	26.7335	26.4120	NA	29.4702	29.1073	29.0786	-2.6459	2.0692		
	**91**	30.3039	30.2389	NA	32.6392	32.5304	NA	-2.3134	1.6433		
	**40**	26.5364	26.7241	26.5116	28.6134	28.2939	28.3397	-1.8249	1.1713		
	**64**	29.7344	29.8282	29.7491	28.9487	29.0682	29.3106	0.6614	0.2090		
	**65**	27.8944	28.1979	28.6194	28.6622	29.1533	28.9097	-0.6712	0.5264		
	**72**	NA	25.6592	25.3838	26.8240	27.2693	26.9823	-1.5037	0.9375		
	**38**	28.6419	28.6979	28.3159	29.0247	29.0384	29.1770	-0.5282	0.4768		
	**41**	27.1881	NA	27.0604	28.1976	28.2304	28.0943	-1.0498	0.6844		
	**69**	29.3052	29.2352	29.0552	31.1042	31.4742	31.5442	-2.1757	1.4937		
	**73**	27.2252	27.0552	26.9052	29.4942	29.6542	NA	-2.5123	1.8863		
	**74**	26.4052	26.4152	26.3652	28.4442	28.5942	28.8242	-2.2257	1.5463		
	**66**	25.5003	25.1074	25.6856	26.1381	26.0750	26.0910	-0.6703	0.5261		
**HC (N = 30)**	**67**	NA	25.1159	24.8405	26.9127	27.2019	27.2242	-2.1347	1.4518	1.0000 ± 0.11	
	**78**	25.9721	25.9483	26.1576	26.9127	27.2019	27.2242	-1.0869	0.7023		
	**68**	24.8365	24.5759	24.5200	26.9148	NA	27.3448	-2.4857	1.8517		
	**75**	29.7278	29.7565	NA	31.3110	31.1863	31.1434	-1.4714	0.9167		
	**87**	30.2264	30.3724	30.1543	31.7620	31.8816	NA	-1.5708	0.9821		
	**89**	28.7634	NA	28.5250	29.5493	29.7278	NA	-0.9943	0.6586		
	**79**	26.8447	26.5841	26.5283	26.2643	26.3235	26.3273	0.3473	0.2599		
	**81**	27.9611	27.6043	27.6370	27.4516	27.6737	26.5745	0.5009	0.2336		
	**82**	25.3587	25.3033	25.3108	26.2184	26.1892	26.3238	-0.9195	0.6253		
	**83**	22.0879	22.0055	22.1769	24.1230	24.2570	24.0869	-2.0656	1.3839		
	**84**	26.6360	27.2757	26.6801	26.3278	26.4012	25.9368	0.6420	0.2119		
	**88**	26.7741	26.8684	NA	28.8541	28.8239	28.8004	-2.0049	1.3269		
	**86**	25.3043	25.3164	25.2785	26.3879	26.5059	26.1083	-1.0343	0.6771		
	**77**	29.3286	29.0986	29.0986	31.4276	30.6876	NA	-1.8823	1.2189		
	**85**	29.5186	NA	29.4286	30.2376	30.2776	NA	-0.7840	0.5693		
	**80**	NA	24.7369	24.6869	27.5542	NA	27.3042	-2.7173	2.1743		
